# Supplementary material for: BnKAT2 Positively Regulates the Main Inflorescence Length and Silique Number in Brassica napus by Regulating the Auxin and Cytokinin Signaling Pathways
Source: Plants (Basel). 2022 Jun 24;11(13):1679. doi: 10.3390/plants11131679 (PMC9269334; doi:10.3390/plants11131679)
Supplement: Supplementary file 1 [file plants-11-01679-s001.zip › plants-1753466-supplementary.pdf]

Table S1. DEGs involved in plant hormone signaling pathways

Up-/downregulated in the *BnKAT2*- overexpressing *B. napus* plants	Gene ID	Putative *A. thaliana* homolog	Annotation
Downregulated	BnaA06g06240D	AT1G10470	ARR4
	BnaC08g27970D	AT3G57040	ARR9
	BnaA06g22370D	AT5G62920	ARR6
	novel.20	AT3G48100	ARR5
	BnaA03g19410D	AT2G41310	ARR3
	novel.1210	AT3G48100	ARR5
	BnaC05g14720D	AT1G19050	ARR7
	BnaC06g22690D	AT1G74950	TIFY10B
	BnaC06g23450D	AT1G73730	EIL3
	BnaA01g30760D	AT3G12250	TGA6
	BnaA05g32040D	AT3G05120	GID1A alpha/beta-Hydrolases superfamily protein
	BnaCnng41350D	AT1G04250	Aux/IAA transcriptional regulator family protein
	novel.2588	AT4G39400	Leucine-rich receptor-like protein kinase family protein
	BnaC07g21260D	AT2G01830	CHASE domain containing histidine kinase protein
	BnaC06g43070D	AT1G80100	Histidine phosphotransfer protein 6
	BnaC01g22110D	AT1G27320	Histidine kinase 3
	BnaA09g16590D	AT5G43700	Aux/IAA transcriptional regulator family protein-2
	BnaA01g33540D	AT3G04580	Signal transduction histidine kinase
Upregulated	BnaAnng23920D	AT3G11410	PP2CA
	BnaA05g32630D	AT3G03450	RGL2
	novel.1470	AT1G27320	HK3
	BnaC09g34350D	AT5G59220	PP2C protein
	BnaA09g44210D	AT1G19350	Brassinosteroid signaling positive regulator (BZR1) family protein
	BnaC03g50700D	AT5G63650	SNF1-related protein kinase 2.5
